# Determinants of bone mineral density in various regions of the skeleton among smokers and non-smokers: the role of physical activity

**DOI:** 10.3389/fphys.2024.1403102

**Published:** 2024-09-19

**Authors:** Anna Kopiczko, Michał Czapla, Grzegorz Kubielas, Bartosz Uchmanowicz

**Affiliations:** ^1^ Department of Human Biology, Józef Piłsudski University of Physical Education in Warsaw, Warszawa, Poland; ^2^ Division of Scientific Research and Innovation in Emergency Medical Service, Department of Emergency Medical Service, Faculty of Nursing and Midwifery, Wroclaw Medical University, Wroclaw, Poland; ^3^ Institute of Heart Diseases, University Hospital, Wroclaw, Poland; ^4^ Group of Research in Care (GRUPAC), Faculty of Health Science, University of La Rioja, Logrono, Spain; ^5^ Division of Healthcare Organisation, Department of Nursing, Faculty of Nursing and Midwifery, Wroclaw Medical University, Wroclaw, Poland

**Keywords:** bone mineral density, various regions of skeleton, Caucasian ethnic group, smoking, physical activity

## Abstract

**Background:**

The adult human skeleton is composed of cortical and cancellous bone. The proportions of these two types of bone tissue differ in various parts of the skeleton. The aim of this cross-sectional study was to quantify the determinants of bone mineral density (BMD) and bone mineral content in various regions of interest (ROIs) in smokers and never-smokers.

**Methods:**

In this study, 4,332 bone scans of three regions of interest (ROIs) were analyzed: the forearm (distal and proximal), femur, and lumbar spine. Body composition and bone parameters were measured using dual-energy X-ray absorptiometry. Smoking was measured using the Global Adult Tobacco Survey questionnaire. Body mass index (BMI) was calculated, and physical activity (PA) was characterized by the metabolic equivalent of task (MET).

**Results:**

Among women, the interaction between PA (positive β coefficient) and smoking (negative β coefficient) was a significant predictor of BMD in the distal and proximal forearm (adj. R^2^ = 0.40 and R^2^ = 0.58; *p* < 0.001). The interaction of three variables—age, smoking (negative β), and MET (positive β)—was significant for total hip BMD (adj. R^2^ = 0.54; *p* < 0.001). The interaction between BMI and MET (positive β) and smoking (negative β) was significant for BMD in the lumbar spine (adj. R^2^ = 0.62; *p* < 0.001). In men, the interaction between MET (positive β) and smoking (negative β) was significant for BMD in the forearm and lumbar spine (adj. R^2^ = 0.44, R^2^ = 0.46, and R^2^ = 0.49; *p* < 0.01). Smoking alone was a significant negative predictor of total hip BMD (adj. R^2^ = 0.34; *p* < 0.001).

**Conclusion:**

Among both women and men, never-smokers had significantly better bone parameters than smokers. Smoking was a significant negative predictor for BMD in the various ROIs in both women and men. Physical activity was a significant positive predictor of BMD, with a strong association with bone parameters.

## Background

The adult human skeleton is composed of cortical and cancellous bone. The proportions of these two types of bone tissue differ in various parts of the skeleton. Cancellous bone predominates in the vertebrae, whereas long bones contain mostly cortical bone (Compston et al., 2019). Bone remodeling is a process by which old bone is replaced with new bone. During remodeling, voids and osteoid form temporarily because bone resorption and osteoid formation precede mineralized bone formation. The process of mineralization in the bone depends on the rate of bone turnover. A slower rate of bone turnover allows bone to accumulate more minerals before being resorbed in a successive remodeling cycle (Hernandez et al., 2001). Values of bone mineral density (BMD) and bone mineral content (BMC) are used to diagnose bone health. BMD is conditioned by many factors, including genetic predisposition. However, more and more research is focused on identifying modifiable lifestyle factors.

The current research shows that smoking may have detrimental effects on the skeletal system. Recent evidence demonstrates that tobacco smoking causes an imbalance in the mechanisms of bone turnover, leading to lower BMC and BMD, thus significantly increasing the risk of osteoporosis and fractures (Wong et al., 2007; Ward and Klesges, 2001a; Al-Bashaireh et al., 2018). However, reports on the association between cigarette smoking and BMD in men and women are contradictory and often affect only one location of the skeleton, axial or peripheral. From a population health perspective, it is important to know the determinants of BMD in smokers and to identify variables that can help repair the harmful effects of smoking on bone health.

Currently, the high quality of bone tissue measurements allows for detailed determination of mineral density at specific regions of interest (ROIs). The most common sites for measurements are the lumbar spine, proximal femur, forearm, and calcaneus. Each ROI correlates moderately well with the others and provides a reasonable prediction of the risk of generic osteoporosis and fractures. However, this correlation does not necessarily lead to consistent diagnostic classification of an individual when threshold-based criteria are used (Lu et al., 2001). Bone tissue is a living and active tissue. An analysis of the effect of multiple variables on bone density simultaneously in several ROIs can help further investigate their interactions and their mechanisms.

Therefore, in this cross-sectional study on a population-based cohort of adult Caucasian women and men of European origin, we aimed to quantify the determinants of bone mineral density and bone mineral content in various regions of interest among smokers and never-smokers.

## Material and methods

### Study population

This cross-sectional study comprised 4,332 bone scans of men (aged 39.3 ± 13.1 years) and women (aged 44.8 ± 12.5 years) from the Polish population (Caucasian ethnic group, of European origin), divided into three groups according to ROI: forearm (distal and proximal), femur, and lumbar spine. We analyzed 2,646 bone scans from never-smokers and 1,692 from smokers (current or former) at selected ROIs (Table 1).

**TABLE 1:** Number of DXA scans (n) in the various regions of interest (ROIs): forearm, femur, and lumbar spine across the groups and by smoking status.

ROIs	All scans (n) Women/Men	Never-smokers (n) Women/Men	Smokers (n) Women/Men
Forearm Distal radius BMD (g/cm^2^) Distal radius BMC (g)	588 308/280	322 143/179	266 165/101
Proximal radius BMD (g/cm^2^) Proximal radius BMC (g)	588 308/280	322 143/179	266 165/101
Femur Femoral neck BMD (g/cm^2^) Femoral neck BMC (g)	454 325/129	279 211/68	175 114/61
Troch BMD (g/cm^2^) Troch BMD BMC (g)	454 325/129	279 211/68	175 114/61
Total hip BMD (g/cm^2^) Total hip BMC (g)	454 325/129	279 211/68	175 114/61
Lumbar spine L1 BMD (g/cm^2^) L1 BMC (g)	360 262/98	233 167/66	127 95/32
L2 BMD (g/cm^2^) L2 BMC (g)	360 262/98	233 167/66	127 95/32
L3 BMD (g/cm^2^) L3 BMC (g)	360 262/98	233 167/66	127 95/32
L4 BMD (g/cm^2^) L4 BMC (g)	360 262/98	233 167/66	127 95/32
Total lumbar spine BMD (g/cm^2^) Total lumbar spine BMC (g)	360 262/98	233 167/66	127 95/32
Total scanned ROIs	**4338** **2901/1437**	**2646** **1754/892**	**1692** **1147/545**

Legend for this table: BMD, bone mineral density; BMC, bone mineral content; ROIs, regions of interest.

The project was carried out in Warsaw, the capital of Poland. Recruitment for participation in the study was continuous. The criteria for participation included being an adult not diagnosed with or undergoing pharmacological treatment for chronic diseases and providing written consent. In the first stage, a health interview was conducted with each applicant. Subjects with contraindications to densitometric examination were excluded. The exclusion criteria also included hormone replacement therapy in women, kidney disease, cancers, rheumatoid arthritis, bone diseases, nutritional disorders, and long-term steroid treatment. All participants were informed about the aims and schedule of the study and received their test results along with an interpretation.

### Measurement of body composition and bone parameters

Body composition and bone parameters were measured using dual-energy X-ray absorptiometry (DXA) with a Norland XR-46 bone densitometer equipped with K edge filtering systems (Swissray-USA, Norland Medical Systems, Madison, WI, United States). Fat mass (FM), fat-free mass (FFM), bone mineral density (BMD), bone mineral content (BMC), and T-scores were measured. This study used the following ROIs for bone parameters: lumbar spine L1–L4, proximal femur (femoral neck, trochanter, and total hip), and distal and proximal parts of the forearm. The long-term precision error for a reference lumbar spine phantom expressed as a percentage coefficient of variance was less than 1% during the study period. All subjects in this study received total and subregional DXA scans. DXA scans were performed by certified radiology technicians. The long-term stability of the densitometer was assessed by daily phantom scans, as recommended by the manufacturer, and calibration standards. A standardized procedure for positioning and data analysis was established and followed for all scans. The effective dose (μSv) for DXA scans of the forearm was less than 1 μSv. The lumbar spine, hip, and whole-body scans each resulted in an effective dose of approximately 1 μSv. The body mass index (BMI) was calculated.

### Smoking status and covariates

Smoking was measured with the Global Adult Tobacco Survey questionnaire (GATS), section B, on active smoking (AS). A direct interview was conducted by a trained interviewer with extensive experience in collecting data using this method and the GATS questionnaire. The survey was conducted following the guidelines of WHO experts and the GATS methodology used in Poland, including a standard protocol for the interview questionnaires, sample weights, data management, analysis, reporting, and information release. In Poland, the Ministry of Health revised and approved the questionnaires and also appointed two committees—the GATS Poland Scientific Committee and the GATS Poland Steering Committee—that handle the scientific and technical coordination of the nationally representative GATS survey (Kaleta et al., 2009). The following data were collected: smoking status (never-smokers/smokers), smoking duration (number of years of smoking), the number of cigarettes smoked per day, and pack-years of smoking.

### Assessment of physical activity

The International Physical Activity Questionnaire (IPAQ) was used to assess the present level of physical activity in a direct interview, as recommended in international studies such as the European Health Interview Survey (EUROHIS) and the European Physical Activity Surveillance System (EUPASS). Weekly physical activity was calculated by adding up the values for the metabolic equivalent of task (MET, min/week) obtained during vigorous activity, moderate activity, and walking performed throughout the entire week (Lee et al., 2011).

### Statistical analysis

All statistical analyses were conducted using STATISTICA software (v.12, StatSoft, United States). The normality of the data distribution was assessed using the Shapiro–Wilk test for each variable. In cases where the data were not normally distributed, the Box–Cox transformation was applied. Effect sizes were calculated using Cohen’s d = 2t//(df^1/2^), (small effect: <0.5; medium effect: 0.5–0.8; large effect: >0.8). To assess the impact of selected variables on BMD and BMC, an analysis of covariance (ANCOVA) was performed. The analysis included covariates such as age, body mass index (BMI), fat mass (FM), fat-free mass (FFM), number of cigarettes smoked per day, and physical activity level measured by the metabolic equivalent of task (MET). Multiple regression analysis was used to identify predictors of BMD and BMC in several ROIs. Statistical significance was set at *p* < 0.01 and *p* < 0.001 for all analyses.

## Results

### Characteristics of the study participants

Table 2 presents the baseline characteristics of the study group by various ROIs, sex, and smoking status. The analysis of particular variables among the women, taking into account smoking status, showed several significant differences. Significantly higher values (*p* < 0.001) of all bone parameters (BMD, BMC, and T-score) in all ROIs, as well as MET (min/week), were noted among female never-smokers than among smokers (large effect). Among the group with forearm ROI, the female never-smokers had significantly higher body weight (3.3 kg higher; *p* < 0.01; small effect) and fat-free mass (2.9 kg; *p* < 0.001; medium effect) than the smokers. In the group with the forearm ROI, the female never-smokers had significantly higher fat-free mass (1.6 kg higher; *p* < 0.001; small effect) than the smokers. In the group with the femur ROI, the female never-smokers had significantly higher fat mass (5.3 kg higher; *p* < 0.001; medium effect) and significantly lower fat-free mass (2.1 kg lower; *p* < 0.01; medium effect) than the smokers.

**TABLE 2:** Baseline characteristics of the study population by various regions of interest (ROIs) and smoking status.

	Women never-smokers	Women smokers	*Cohen’s d*	Men never-smokers	Men smokers	*Cohen’s d*
Mean ± SD						
Forearm	N = 143	N = 165		N = 179	N = 101	
Age (years)	30.0 ± 5.0	31.3 ± 4.4	0.277	29.9 ± 4.5	30.9 ± 4.7	0.217
Body weight (kg)	65.5 ± 10.3	62.2 ± 10.5*	0.317	82.3 ± 10.7	75.8 ± 8.0**	0.695
FM (kg)	19.2 ± 7.4	18.8 ± 6.9	0.056	17.0 ± 5.3	14.5 ± 3.4**	0.575
FFM (kg)	46.3 ± 4.3	43.4 ± 4.6**	0.652	65.3 ± 6.3	61.2 ± 5.5**	0.695
BMI (kg/m^2^)	22.6 ± 3.5	22.7 ± 3.9	0.027	24.8 ± 2.6	23.4 ± 2.2**	0.583
Ultradis. BMD (g/cm^2^)	0.407 ± 0.06	0.325 ± 0.06**	1.367	0.514 ± 0.01	0.427 ± 0.01**	8.700
Ultradis. BMC (g)	1.585 ± 0.24	1.283 ± 0.20**	1.373	2.257 ± 0.33	1.930 ± 0.28**	1.072
Prox. BMD (g/cm^2^)	0.798 ± 0.07	0.646 ± 0.09**	1.900	0.956 ± 0.11	0.794 ± 0.10**	1.543
Prox. BMC (g)	2.085 ± 0.28	1.699 ± 0.25**	1.457	2.879 ± 0.36	2.392 ± 0.30**	1.476
MET (min/week)	1,086.1 ± 564.0	300.9 ± 307.4**	1.802	948.6 ± 624.4	275.5 ± 186.9**	1.659
Femur	N = 211	N = 114		N = 68	N = 61	
Age (years)	49.7 ± 9.0	51.3 ± 9.1	0.177	50.1 ± 10.9	52.4 ± 10.6	0.214
Body weight (kg)	68.9 ± 12.6	65.9 ± 10.4	0.261	88.5 ± 15.4	83.7 ± 12.9	0.339
FM (kg)	25.7 ± 9.5	24.3 ± 7.6	0.164	25.5 ± 9.8	23.8 ± 7.5	0.197
FFM (kg)	43.2 ± 4.4	41.6 ± 4.0**	0.381	63.0 ± 7.3	59.8 ± 6.7*	0.457
BMI (kg/m^2^)	25.2 ± 4.6	24.8 ± 4.3	0.090	27.3 ± 4.1	26.7 ± 3.6	0.156
Fem. neck BMD (g/cm^2^)	0.92 ± 0.13	0.74 ± 0.1**	1.565	1.02 ± 0.15	0.85 ± 0.11**	1.354
Fem. neck BMC (g)	4.50 ± 0.76	3.70 ± 0.54**	1.217	5.66 ± 1.24	4.78 ± 0.65**	0.929
Troch BMD (g/cm^2^)	0.74 ± 0.12	0.62 ± 0.09**	1.190	0.90 ± 0.14	0.78 ± 0.10**	1.042
Troch BMC (g)	9.21 ± 2.27	7.65 ± 1.50**	0.828	14.10 ± 3.47	12.38 ± 2.72*	0.557
Total hip BMD (g/cm^2^)	0.97 ± 0.12	0.81 ± 0.11**	1.365	1.11 ± 0.16	0.95 ± 0.10**	1.231
Total hip BMC (g)	31.60 ± 4.77	26.73 ± 3.64**	1.158	41.84 ± 6.81	36.01 ± 4.41**	1.039
MET (min/week)	679.3 ± 450.9	161.5 ± 124.2**	1.801	819.8 ± 563.3	124.3 ± 185.3**	1.858
Lumbar spine	N = 167	N = 95		N = 66	N = 32	
Age (years)	52.8 ± 9.1	54.7 ± 11.0	0.189	49.4 ± 12.6	50.0 ± 10.8	0.051
Body weight (kg)	69.3 ± 12.7	66.2 ± 11.3	0.258	83.8 ± 9.0	81.2 ± 11.2	0.257
FM (kg)	30.3 ± 8.9	25.0 ± 8.3**	0.616	22.2 ± 5.9	22.3 ± 7.3	0.015
FFM (kg)	39.0 ± 7.4	41.1 ± 4.3*	0.359	61.7 ± 5.7	58.9 ± 6,1	0.475
BMI (kg/m^2^)	25.4 ± 4.8	24.9 ± 4.3	0.110	25.8 ± 2.6	26.4 ± 3.4	0.200
L1 BMD (g/cm^2^)	1.12 ± 0.17	0.84 ± 0.12**	1.959	1.18 ± 0.191	0.92 ± 0.11**	1.728
L1 BMC (g)	14.89 ± 3.69	11.67 ± 2.74**	1.000	18.96 ± 6.78	14.81 ± 3.46*	0.811
L2 BMD (g/cm^2^)	1.16 ± 0.16	0.87 ± 0.12**	2.071	1.25 ± 0.24	0.96 ± 0.12**	1.510
L2 BMC (g)	16.44 ± 3.16	12.44 ± 2.01**	1.549	21.02 ± 5.486	15.68 ± 2.94**	1.268
L3 BMD (g/cm^2^)	1.17 ± 0.17	0.90 ± 0.12**	1.910	1.25 ± 0.20	0.99 ± 0.14**	1.503
L3 BMC (g)	17.71 ± 3.47	13.51 ± 2.12**	1.502	23.26 ± 6.26	17.62 ± 2.40**	1.303
L4 BMD (g/cm^2^)	1.16 ± 0.16	0.89 ± 0.13**	1.869	1.27 ± 0.18	0.96 ± 0.14**	1.962
L4 BMC (g)	19.06 ± 3.65	13.88 ± 2.67**	1.640	25.12 ± 6.78	17.47 ± 2.87**	1.585
Total LS BMD (g/cm^2^)	1.24 ± 0.16	0.94 ± 0.12**	2.157	1.34 ± 0.20	1.00 ± 0.15**	1.892
Total LS BMC (g)	73.5 ± 13.7	55,42 ± 7.9**	1.676	95.25 ± 23.07	68.42 ± 12.11**	1.525
MET (min/week)	703.2 ± 270.2	312.7 ± 255.8**	1.485	906.1 ± 609.6	340.8 ± 369.4**	1.155

Legend for this table: BMD, bone mineral density; BMC, bone mineral content; BMI, body mass index; FM, fat mass; FFM, fat-free mass; MET, metabolic equivalent of task; d, effect sizes calculated using Cohen’s formula; the levels of statistical significance *p-value*: *p < 0.01; **p < 0.001.

Similar analyses were carried out in the group of men. As in the group of women, significantly higher values (*p* < 0.01 and *p* < 0.001) of all bone parameters (BMD, BMC, and T-score) in all ROIs, as well as MET (min/week), were noted in the male never-smokers than in the smokers (in all ROIs, there were large effects except for trochanter BMC, in which there was a small effect). In the group with the forearm ROI, the male never-smokers had significantly higher levels of all somatic parameters (*p* < 0.001; medium effect) than the smokers. In the group with the femur ROI, the male never-smokers had significantly higher fat-free mass (3.2 kg higher; *p* < 0.01; medium effect) than the smokers (Table 2).

### Determinants of BMD in the various regions of interest

The results of the covariance analysis (ANCOVA) between BMD in the various ROIs and selected parameters are presented in Table 3. Covariance analysis indicated that, in the women, the main parameters that significantly impact BMD in the distal forearm were MET (min/week) and cigarettes/day (adj.R^2^ = 0.43). In contrast, physical activity significantly influenced BMD in the proximal forearm and total hip (adj. R2 = 0.60, adj. R2 = 0.56). Fat-free mass and MET significantly influenced the BMD of the lumbar spine (adj. R^2^ = 0.65). Similarly, the analysis of the men indicated that the main parameters that significantly influenced the BMD of the distal forearm were BMI, FM, FFM, and MET (adj. R^2^ = 0.48). Physical activity significantly influenced BMD in the proximal forearm and lumbar spine (adj. R2 = 0.46; adj. R2 = 0.51) (Table 3).

**TABLE 3:** Relationships between BMD (g/cm^2^) in the various regions of interest (ROIs) and BMI, body composition, physical activity, and smoking status in men and women (results of ANCOVA, F test, p).

	BMD Ultradis. forearm	BMD Prox. forearm	BMD Total hip	BMD total lumbar spine
	*F(p)*	*F(p)*	*F(p)*	*F(p)*
Women				
Age (years)	0.233 (0.630)	0.339 (0.561)	4.392 (0.037)	2.684 (0.103)
BMI (kg/m^2^)	1.323 (0.251)	0.101 (0.751)	0.009 (0.926)	0.614 (0.434)
FM (kg)	2.315 (0.129)	0.176 (0.675)	0.636 (0.426)	0.338 (0.561)
FFM (kg)	0.229 (0.632)	0.021 (0.886)	0.196 (0.658)	14.801 (0.000)**
MET (min/week)	40.27 (0.000)**	34.89 (0.000)**	84.46 (0.000)**	50.628 (0.000)**
Smoking duration (years)	0.295 (0.588)	0.282 (0.596)	0.477 (0.490)	0.008 (0.928)
Cigarettes/day (n)	4.970 (0.011)**	0.215 (0.643)	0.102 (0.750)	0.044 (0.833)
Pack-year smoking (n)	0.319 (0.572)	2.736 (0.099)	2.261 (0.134)	0.591 (0.443)
Smoking status	1.789 (0.182)	1.361 (0.244)	0.116 (0.734)	0.565 (0.453)
F(p) R^2^ adj	27.08 (0.000) 0.43	51.14 (0.000) 0.60	46.24 (0.000) 0.56	60.70 (0.000) 0.65
Men				
Age (years)	2.755 (0.098)	1.716 (0.191)	0.709 (0.401)	1.456 (0.231)
BMI (kg/m^2^)	6.441 (0.012)**	0.314 (0.576)	0.045 (0.833)	1.187 (0.279)
FM (kg)	5.961 (0.013)**	1.243 (0.266)	0.006 (0.937)	2.600 (0.110)
FFM (kg)	6.087 (0.014)**	1.039 (0.309)	2.069 (0.150)	0.930 (0.338)
MET (min/week)	90.60 (0.00)**	22.17 (0.000)**	0.964 (0.328)	4.386 (0.011)**
Smoking duration (years)	1.914 (0.169)	0.325 (0.569)	0.065 (0.799)	0.365 (0.417)
Cigarettes/day (n)	0.263 (0.609)	0.558 (0.456)	1.295 (0.257)	0.666 (0.417)
Pack-year smoking (n)	3.122 (0.078)	2.340 (0.127)	0.005 (0.946)	0.103 (0.749)
Smoking status	0.310 (0.578)	0.744 (0.389)	0.464 (0.497)	0.075 (0.785)
F(p) R^2^ adj	29.02 (0.000) 0.48	27.61 (0.000) 0.46	9.86 (0.000) 0.38	12.22 (0.000) 0.51

Legend for this table: BMD, bone mineral density; BMI, body mass index; FM, fat mass; FFM, fat-free mass; MET, metabolic equivalent of task; F, Ronald A. Fisher’s test; p, *p*-value; level of statistical significance *p < 0.05 and **p < 0.01; Rˆ2 adj., the adjusted R-squared values of determination.

### Determinants of BMC in the various regions of interest

The results of the covariance analysis (ANCOVA) between BMC in the various ROIs and selected parameters are presented in Table 4. The ANCOVA indicated that, in the women, the main parameters that significantly influenced BMC in the distal forearm were FFM, MET, and cigarettes/day (adj. R^2^ = 0.46). FFM and MET influenced BMC in the proximal forearm and total hip (adj. R^2^ = 0.50; adj. R^2^ = 0.48). Four variables, BMI, FM, FFM, and MET, significantly influenced the BMC lumbar spine (adj. R2 = 0.54). Similarly, the analysis of the men indicated that the two parameters, FFM and MET, influenced BMC in the distal forearm (adj. R^2^ = 0.36). In contrast, only physical activity influenced BMD in the proximal forearm (adj. R2 = 0.52). Two variables, FFM and the number of cigarettes smoked per day, influenced the BMC of the total hip (adj. R2 = 0.44) (Table 4).

**TABLE 4:** Relationships between BMC (g) in the various regions of interest (ROIs) and BMI, body composition, physical activity, and smoking status in men and women (results of ANCOVA, F test, p).

	BMC Ultradis. Forearm	BMC Prox. Forearm	BMC Total hip	BMC total lumbar spine
	*F(p)*	*F(p)*	*F(p)*	*F(p)*
Women				
Age (years)	0.299 (0.585)	1.474 (0.226)	0.005 (0.945)	1.677 (0.196)
BMI (kg/m^2^)	2.237 (0.136)	2.267 (0.133)	0.413 (0.521)	20.560 (0.000)**
FM (kg)	5.881 (0.016)	2.238 (0.136)	0.794 (0.374)	17.099 (0.000)**
FFM (kg)	16.59 (0.000)**	9.097 (0.003)**	15.666 (0.000)**	63.559 (0.000)**
MET (min/week)	16.92 (0.000)**	37.39 (0.000)**	28.395 (0.000)**	26.463 (0.000)**
Smoking duration (years)	4.271 (0.040)	0.242 (0.623)	0.007 (0.936)	0.134 (0.714)
Cigarettes/day (n)	6.227 (0.013)**	1.466 (0.227)	0.031 (0.861)	0.000 (0.987)
Pack-year smoking (n)	2.696 (0.102)	0.003 (0.960)	0.428 (0.513)	0.001 (0.981)
Smoking status	3.795 (0.052)	0.484 (0.487)	0.037 (0.847)	0.621 (0.432)
F(p) R^2^ adj	29.65 (0.000) 0.46	35.01 (0.000) 0.50	33.78 (0.000) 0.48	34.62 (0.000) 0.54
Men				
Age (years)	3.321 (0.069)	0.163 (0.687)	2.940 (0.089)	0.001 (0.983)
BMI (kg/m^2^)	4.621 (0.032)	0.018 (0.894)	1.882 (0.173)	1.023 (0.315)
FM (kg)	3.419 (0.066)	0.001 (1.002)	0.488 (0.486)	0.813 (0.370)
FFM (kg)	10.65 (0.001)**	5.437 (0.020)	7.538 (0.007)**	1.123 (0.292)
MET (min/week)	23.22 (0.000)**	39.52 (0.000)**	2.965 (0.088)	0.842 (0.361)
Smoking duration (years)	1.320 (0.252)	0.203 (0.653)	0.065 (0.799)	0.126 (0.724)
Cigarettes/day (n)	0.848 (0.358)	0.247 (0.620)	6.654 (0.011)**	0.001 (0.979)
Pack-year smoking (n)	2.965 (0.086)	0.179 (0.672)	2.543 (0.113)	0.327 (0.569)
Smoking status	0.751 (0.387)	0.254 (0.614)	5.367 (0.022)	0.167 (0.684)
F(p) R^2^ adj.	18.46 (0.000) 0.36	35.14 (0.000) 0.52	12.25 (0.000) 0.44	6.42 (0.000) 0.33

Legend for this table: BMD, bone mineral density; BMI, body mass index; FM, fat, mass; FFM, fat-free mass; MET, metabolic equivalent of task; F, Ronald A. Fisher’s test; p, *p*-value; level of statistical significance *p < 0.05 and **p < 0.01; Rˆ2 adj., the adjusted R-squared values of determination.

### Results of the multiple regression analysis

The results of the multiple regression analysis are presented in Tables 5, 6. The model explains 34%–62% (adj. R^2^ = 0.34–0.62) of the variance in BMD determinants in four ROIs (distal forearm, proximal forearm, total hip, and lumbar spine) in both men and women. Among the women, the predictors of interactions of physical activity (positive direction of β coefficient) and smoking (negative direction) were significant for BMD of the distal and proximal forearm (adj. R^2^ = 0.40 and R^2^ = 0.58; *p* < 0.001). It was also found that the predictors of interactions of three variables—age, smoking (negative directions), and MET (positive direction)—were significant for total hip BMD (adj. R^2^ = 0.54; *p* < 0.001). Furthermore, the predictors of interactions of BMI and MET (positive direction) and smoking (negative direction) were significant for BMD of the total lumbar spine (adj. R^2^ = 0.62; *p* < 0.001). Among the men, the predictors of interactions of physical activity (positive direction) and smoking (negative direction) were significant for BMD in the two forearm ROIs and the lumbar spine (adj. R^2^ = 0.44, R^2^ = 0.46, R^2^ = 0.49; *p* < 0.01). Smoking itself was a significant predictor (negative value of the standardized β coefficient) for total hip BMD (adj. R^2^ = 0.34; *p* < 0.001) (Table 5).

**TABLE 5:** Determinant of BMD (g/cm^2^) in the various regions of interest (ROIs)—results of multiple regression.

Determinant	BMD Ultradis. Forearm	BMD Prox. Forearm	BMD Total hip	BMD total lumbar spine
	β ± SE	β ± SE	β ± SE	β ± SE
Women				
Age	0.021 ± 0.054	0.044 ± 0.045	−0.158 ± 0.045**	0.092 ± 0.042
BMI	0.128 ± 0.160	0.001 ± 0.133	0.002 ± 0.156	0.300 ± 0.070**
FM	−0.183 ± 0.162	−0.016 ± 0.135	0.218 ± 0.161	−0.142 ± 0.071
Smoking	−0.288 ± 0.065**	−0.543 ± 0.054**	−0.333 ± 0.045**	−0.526 ± 0.053**
MET	0.418 ± 0.060**	0.313 ± 0.050**	0.418 ± 0.046**	0.383 ± 0.052**
R^2^ adj	0.40	0.58	0.54	0.62
Men				
Age	−0.017 ± 0.052	−0.051 ± 0.051	−0.056 ± 0.091	−0.042 ± 0.114
BMI	0.166 ± 0.127	0.028 ± 0.125	−0.450 ± 0.316	−0.259 ± 0.281
FM	−0.102 ± 0.129	0.059 ± 0.128	0.676 ± 0.328	0.506 ± 0.315
Smoking	−0.225 ± 0.054**	−0.479 ± 0.053**	−0.418 ± 0.093**	−0.559 ± 0.086**
MET	0.502 ± 0.053**	0.255 ± 0.052**	0.139 ± 0.091	0.201 ± 0.088*
R^2^ adj	0.44	0.46	0.34	0.49

Legend for this table: BMD, bone mineral density; BMI, body mass index; FM, fat mass; MET, metabolic equivalent of task.

β ± SE, values are regression coefficients ±SE, describing the change in BMD (g/cm^2^) associated with age, BMI, FM, and smoking status.

R^2^ adj., coefficient of determination: a measure of the proportion of variation in BMD, explained by the variation in the risk factors. Statistical significance was set at the levels of *p < 0.01 and **p < 0.001.

**TABLE 6:** Determinant of BMC (g) in the various regions of interest (ROIs)—results of multiple regression.

Determinant	BMC Ultradis. Forearm	BMC Prox. Forearm	BMC Total hip	BMC total lumbar spine
	β ± SE	β ± SE	β ± SE	β ± SE
Women				
Age	−0.061 ± 0.053	0.005 ± 0.051	−0.206 ± 0.049**	0.025 ± 0.052
BMI	0.048 ± 0.157	0.073 ± 0.150	−0.520 ± 0.171**	0.092 ± 0.086
FM	−0.058 ± 0.160	0.030 ± 0.152	0.849 ± 0.176**	0.065 ± 0.088
Smoking	−0.377 ± 0.064**	−0.371 ± 0.061**	−0.319 ± 0.049**	−0.391 ± 0.065**
MET	0.305 ± 0.059**	0.385 ± 0.056**	0.268 ± 0.046**	0.321 ± 0.063**
R^2^ adj	0.42	0.47	0.44	0.42
Men				
Age	−0.043 ± 0.057	−0.098 ± 0.049	−0.099 ± 0.089	−0.226 ± 0.129
BMI	0.004 ± 0.139	−0.206 ± 0.119	−1.387 ± 0.308**	−0.838 ± 0.318*
FM	0.195 ± 0.142	0.385 ± 0.121*	1.543 ± 0.319**	1.089 ± 0.356*
Smoking	−0.288 ± 0.059**	−0.419 ± 0.050**	−0.328 ± 0.091**	−0.434 ± 0.097**
MET	0.279 ± 0.058**	0.312 ± 0.049**	0.180 ± 0.089	0.103 ± 0.100
R^2^ adj	0.33	0.51	0.38	0.34

Legend for this table: BMC, bone mineral content; BMI, body mass index; FM, fat mass; MET, metabolic equivalent of task.

β ± SE, values are regression coefficients ± SE, describing the change in BMC (g) associated with age, BMI, FM, and smoking status.

R^2^ adj., coefficient of determination: a measure of the proportion of variation in BMC, explained by the variation in the risk factors. Statistical significance was set at the levels of *p < 0.01 and **p < 0.001.

The regression analysis model was also used to evaluate the determinants of BMC (Table 6). The model explains 33%–51% (adj. R^2^ = 0.33–0.51) of the variance. Among the women, the predictors of two interactions—physical activity (positive direction) and smoking (negative direction of β coefficient)—were significant for BMD of the distal and proximal forearm as well as the lumbar spine (adj. R^2^ = 0.42, R2 = 0.47, and R^2^ = 42; *p* < 0.001). It was also found that the predictors of interactions of five variables—age, BMI, smoking (negative directions), fat mass, and MET (positive direction)—were significant for total hip BMD (adj. R^2^ = 0.44; *p* < 0.001).

Among men, the predictors of two interactions—physical activity (positive direction) and smoking (negative direction)—were significant for distal BMC (adj. R^2^ = 0.33; *p* < 0.001). It was also found that the predictors of interactions of three variables—smoking (negative directions), fat mass, and MET (positive direction)—were significant for BMC of the proximal forearm (adj. R^2^ = 0.51; *p* < 0.01). Furthermore, the predictors of interactions of BMI and smoking (negative direction) and fat mass (positive direction) were significant for BMC of the total hip and lumbar spine (adj. R^2^ = 0.38, R^2^ = 0.34; *p* < 0.01) (Table 6).

## Discussion

Behavioral medicine indicates an ever-growing interest in the impact of lifestyle behaviors on health (Lopuszanska-Dawid, 2023; Kris-Etherton et al., 2022; Adams et al., 2016). As research indicates, bone tissue health also depends on a number of factors, including lifestyle (Proia et al., 2021; Weaver et al., 2016; Sanchez-Trigo et al., 2022; Watson et al., 2018; Kistler-Fischbacher et al., 2021). Peak bone mass is built up until approximately age 25 (Lu et al., 2016), and this process takes time. Similarly, factors that affect BMD do not show an immediate, short-term impact. Both the building of BMD and the decline of BMD are influenced by many variables over a long period of time. This study evaluated the variables of smoking, smoking duration, and physical activity, that is, determinants that interact in the long term, on BMD.

The study found that, regardless of gender, smokers had significantly lower values of all bone parameters (BMD, BMC, and T-score) in all ROIs, as well as lower MET than never-smokers. The study revealed the most important determinants of BMD in the various ROIs among both women and men. Among women, the main parameters that significantly influenced BMD in the distal forearm were MET and the number of cigarettes per day. Physical activity was the only factor that influenced BMD in the proximal forearm and total hip. Fat-free mass and MET influenced BMD in the lumbar spine. The analysis of the men indicated that the main parameters that significantly influenced BMD in the distal forearm were BMI, FM, FFM, and MET. Physical activity was the only factor that influenced BMD in the proximal forearm and lumbar spine.

Smoking is associated with an increased incidence of fractures and a higher risk of osteoporosis (Vestergaard and Mosekilde, 2003; Yoon et al., 2012), but previous studies have indicated that the mechanism by which smoking affects bone health remains unclear. Additionally, the impact of smoking on bone may differ between men and women (Yoon et al., 2012; Kohler et al., 2021). Smoking can affect bone status either directly or indirectly, but the effect depends on factors such as exposure time to the habit and the number of cigarettes smoked per day (Weng et al., 2022). In this study, the number of years of smoking and pack-years did not significantly affect BMD or BMC, but the number of cigarettes smoked per day did significantly affect BMD and BMC in the distal forearm in women and total hip BMC in men.

In postmenopausal Turkish women, the smokers showed significantly lower vertebra and femur neck BMD than nonsmokers. However, that study did not find a significant relationship between the duration of smoking, the number of cigarettes consumed per day, and BMD (Ugurlu et al., 2016). Another study evaluating the relationship between smoking, the number of cigarettes per day, the length of exposure to the drug, and bone parameters in young women found that BMD did not differ between never-smokers, ex-smokers, and current smokers (Callréus et al., 2013). Some studies have found no significant relationship between smoking and bone mineralization or bone mass (Ward and Klesges, 2001a).

The determinants of BMC were also analyzed. Among women, the main parameters that significantly influenced BMC in the distal forearm were FFM, MET, and cigarettes per day. Two variables—FFM and MET—influenced BMC in the proximal forearm and total hip. Four variables—BMI, FM, FFM, and MET—significantly influenced BMC in the lumbar spine. Similarly, the analysis of men indicated that FFM and MET significantly influenced BMC in the distal forearm. Physical activity was the only factor that influenced BMC in the proximal forearm. Two variables—FFM and the number of cigarettes smoked per day—significantly influenced BMC in the total hip. Recent research has shown that tobacco smoking causes an imbalance in bone turnover, leading to decreased BMC, which makes bones more vulnerable to osteoporosis and fractures. Smoking influences bone mass indirectly by altering body weight, the parathyroid hormone–vitamin D axis, adrenal hormones, and sex hormones, as well as increasing oxidative stress on bone tissue. Additionally, tobacco smoke directly affects BMC by impacting bone osteogenesis and angiogenesis (Al-Bashaireh et al., 2018). Cigarette smoking has deleterious effects on bone integrity. In several studies, a correlation has been shown between declining BMC and the number of cigarettes smoked per day and the years of exposure (Abate et al., 2013; Kanis et al., 2005; Tarantino et al., 2021).

In this study, **for both men and women,** interactions between physical activity (positive influence) and smoking (negative influence) significantly influenced BMD. Physical activity can be an important protective factor in bone health and osteoporosis prevention, as has been shown in many studies (Pinheiro et al., 2020; Ng et al., 2022; Kopiczko, 2020; Kopiczko et al., 2021). Physical activity, especially based on resistance exercises—with weights, multi-directional loading, and weight-bearing—has the most significant physiological effects on bone parameters (Hart et al., 2017; O'Bryan et al., 2022; Kemmler et al., 2020). In several randomized controlled trials, targeted high-impact exercise resulted in increased bone mineral density and bone strength during the prepubertal and peripubertal stages (Ng et al., 2022; Nikander et al., 2010; Hind and Burrows, 2007). Physical activity may be an important factor in protecting bone density in smokers and ex-smokers. Physical activity provides the necessary influence of forces generated from working muscles for proper mineralization. Adequate levels of physical activity affect the normal tissue composition of the body, especially muscle mass and lean body components (Deng et al., 2021; Shin et al., 2011; Lopez et al., 2022; Beaudart et al., 2017; Bao et al., 2020). In this study, FFM also correlated positively with bone parameters in several skeletal regions.

While the detrimental effects of smoking on bone health are well documented, the significance of conducting epidemiological research in this area cannot be overstated (Ward and Klesges, 2001b; Ratajczak et al., 2021). Epidemiological studies provide valuable insights at the population level. By analyzing large cohorts, such as the 4,332 bone scans in our study, we can identify the prevalence and extent of bone density reduction among smokers in a real-world setting. Additionally, such research helps to identify and quantify modifiable risk factors, like smoking and physical activity, that can inform public health interventions. Understanding how these factors interplay allows for developing targeted strategies to mitigate their negative effects. Longitudinal epidemiological studies can help establish causal relationships rather than mere associations (Kundi, 2006). Although our study is cross-sectional, it sets the groundwork for future longitudinal studies that can track changes in bone density over time in relation to smoking behavior. Furthermore, findings from epidemiological studies tend to be more generalizable due to the larger and more diverse populations studied. This enhances the applicability of the results to broader populations, making the conclusions more relevant for public health policies. The data derived from such studies can inform public health policies and smoking cessation programs. By demonstrating the significant negative impact of smoking on bone health, these studies can support legislative measures aimed at reducing smoking prevalence. In conclusion, while the adverse effects of smoking on bone density are well-known, epidemiological research provides a comprehensive understanding of the scale and nuances of this issue. **It underscores** the importance of preventive measures and supports the development of targeted interventions to improve bone health outcomes in the population.

## Conclusion

Our study supports earlier preliminary reports that smoking, particularly prolonged tobacco exposure, results in decreased bone mineralization in various ROIs compared to the never-smoking population. Physical activity can effectively mitigate the development of osteopenia and reduce the heightened risk of osteoporosis in smokers. It is essential to educate young European adults about the adverse effects of smoking, not only on the respiratory system, which is widely recognized, but also on the increased risk of osteoporosis.

## Data Availability

The raw data supporting the conclusions of this article will be made available by the authors, without undue reservation.

## Abbreviations

AS, active smoking; BMC, bone mineral content; BMD, bone mineral density; BMI, body mass index; d effect, sizes calculated using Cohen’s formula; DXA, dual-energy X-ray absorptiometry; EUPASS, European Physical Activity Surveillance System; EUROHIS, European Health Interview Survey; F, Ronald A. Fisher’s test; FFM, fat-free mass; FM, fat mass; GATS, Global Adult Tobacco Survey questionnaire; IPAQ, International Physical Activity Questionnaire; MET, metabolic equivalent of task; PA, physical activity; p-value, level of statistical significance; R^2^ adj, the adjusted R-squared values of determination; ROIs, regions of interest; β ± SE, values are regression coefficients ± SE describing the change in BMD.
